# State Tracking and Fault Diagnosis for Dynamic Systems Using Labeled Uncertainty Graph

**DOI:** 10.3390/s151128031

**Published:** 2015-11-05

**Authors:** Gan Zhou, Wenquan Feng, Qi Zhao, Hongbo Zhao

**Affiliations:** School of Electronic and Information Engineering, Beihang University, Beijing 100191, China; E-Mails: zhouganterry@hotmail.com (G.Z.); buaafwq@buaa.edu.cn (W.F.); bhzhb@buaa.edu.cn (H.Z.)

**Keywords:** dynamic systems, fault diagnosis, concurrent probabilistic automata, Monte Carlo technique, labeled uncertainty graph

## Abstract

Cyber-physical systems such as autonomous spacecraft, power plants and automotive systems become more vulnerable to unanticipated failures as their complexity increases. Accurate tracking of system dynamics and fault diagnosis are essential. This paper presents an efficient state estimation method for dynamic systems modeled as concurrent probabilistic automata. First, the Labeled Uncertainty Graph (LUG) method in the planning domain is introduced to describe the state tracking and fault diagnosis processes. Because the system model is probabilistic, the Monte Carlo technique is employed to sample the probability distribution of belief states. In addition, to address the sample impoverishment problem, an innovative look-ahead technique is proposed to recursively generate most likely belief states without exhaustively checking all possible successor modes. The overall algorithms incorporate two major steps: a roll-forward process that estimates system state and identifies faults, and a roll-backward process that analyzes possible system trajectories once the faults have been detected. We demonstrate the effectiveness of this approach by applying it to a real world domain: the power supply control unit of a spacecraft.

## 1. Introduction

Today’s real-world engineering systems are a product of careful design and manufacturing. These systems usually undergo rigorous testing and validation before deployment. However, due to wear and tear from sustained operations, degradation and faults in system components still occur. In addition, unlikely events and unanticipated situations can also create faults. To avoid these negative effects, it is imperative to accurately track system behavior and timely detect and isolate faults [1,2,3,4].

Model-based diagnosis techniques are frequently used to solve these problems. The key idea is to detect the discrepancies between the actual system behavior and the predictions of a model [5]. Traditionally, two distinct scientific communities have employed different kinds of models to implement their own model-based diagnosis:

- The Fault Detection and Isolation (FDI) methods capture system behavior using differential equation models, whose foundations are based on control and statistical decision theories.
- The Diagnosis (DX) methods use qualitative model and logical approaches, with foundations in the fields of computer science and artificial intelligence.

More specifically, the FDI community has proposed three typical methods to track and diagnose system behavior using the system model: (1) parameter estimation based methods that estimate the value of particular parameter [6]; (2) state estimation based methods that use observers or filters to estimate unknown variables [7]; and (3) parity space based methods that design a set of residuals by eliminating the unknown variables [5]. On the other hand, many researchers in DX community assume that the system can be modeled as a Discrete Event System (DES) [8] at some level of abstraction. A DES model is characterized by a set of discrete states, a set of observable and unobservable events, and transitions between discrete states. The dynamic behavior of DES is described by partitioning time into discrete points at which events occur [9]. On the basis of this system model, the goal of diagnosis is to find unobservable fault events or discrete fault states. Until now, this technique has been widely applied into many domains such as power transmission lines [10] and telecommunication networks [11,12].

Over the years, some researchers and practitioners in both communities are dedicated their efforts to understanding and bridging the FDI and DX approaches. Cordier *et al*. [13] gave a systematic comparison of the analytical redundancy relations (ARR) and conflicts, but their analysis was only applied to the diagnosis of static systems. Bregon *et al*. [14] compared three different structural fault isolation techniques from both communities for linear continuous dynamic systems. Meanwhile, Travé-Massuyès [15] further discussed the facets of diagnosis in the FDI and DX communities, and exemplified how different theories of these areas can be synergistically combined to provide better diagnostic solutions and achieve improved fault management.

In this paper, we only focus on the DX area. In this area, a typical diagnosis approach of DES is based on a diagnoser [16,17], which uses a deterministic finite state machine without emitted events. The diagnosis problem is addressed by compiling the original finite state machine into one that contains only observable transitions and produces the same language in terms of observations. The weakness of this approach is the feasibility of the compilation of the large-scale complex system model into a reasonable size. Some approaches were put forward to overcome this dilemma such as off-line compiler technique [18], distributed diagnosis [19] and hierarchical diagnosis [20].

Simulation-based approach [21,22] is another general method. In this approach, the temporal evolution of system is decomposed into a set of state constraints that hold for the state at each time-step and a set of sequence constraints that restrict the possible transition sequences of such state, so the diagnosis process is performed by checking whether the state constraints and sequence constraints are consistent with the observations. The main problem of this approach is that the number of all possible system trajectories becomes too large to process only after a few time steps.

To address this problem, Williams [21] and Kurien [22] proposed a *k* Best-First Trajectory Enumeration (BFTE). Unfortunately, trajectory probability is significantly underestimated in this approach, because it ignores the additional trajectories that lead to the same state. Considering this shortcoming, Martin [23] presented a *k* Best-First Belief State Enumeration (BFBSE) that increases estimator accuracy and uses less memory and computation time. After that, Williams and Ragno [24] introduced a conflict-based A* (CDA*) search algorithm into these methods, so the belief state search process is further accelerated by eliminating subspaces around each state that are inconsistent with observations. For these *k* best methods, how to choose a suitable *k* value is the key issue. For real-time operation, a large value usually brings more computational complexity, while a small one loses estimator accuracy and even results in misdiagnosis. Moreover, symbolic techniques are also a feasible method. [9] exploited Ordered Binary Decision Diagrams (OBDD) to encode system model and belief state, so the complete belief state can be estimated. However, it still limits its applicability to relatively simple system.

This paper develops a novel approximate simulation-based approach to track both a variety of operational modes of the system and arbitrary combinations of fault conditions, which will enable to determine the most likely system states and trajectories for dynamic diagnosis. First of all, LUG [25] is introduced to describe online monitoring and fault diagnosis of discrete systems. Moreover, the LUG scheme employs the Monte Carlo (MC) technique to sample belief state distribution. The differences between our approach and classical MC technique are twofold. First, the particles in our approach are only focused on sample but not on-line filtering, and particles are assumed to be unit weight throughout simulation. Second, every particle is tagged with unique symbol, so the system evolution trajectories can be easily obtained, and numerous trajectories do not need to be preserved as system evolves. Finally, since Monte Carlo techniques incur sample impoverishment problem, the observation information is combined with prior information to recursively generate the most likely belief states. Although this technique of using observation information has been employed in the literatures [7] and [26], our approach does not need to exhaustively consider all possible successor modes, so it can cope with large number of discrete modes.

The rest of this paper is structured as follows: Section 2 gives some basic definitions about component and system model, simulation-based dynamic diagnosis, and classical belief state update. Section 3 introduces the LUG to describe the dynamic diagnosis process of discrete systems. In Section 4, our approach is formally illustrated and analyzed in detail. Section 5 describes the experimental results on a real-world model: a portion of the power supply control unit of spacecraft. Finally, a conclusion is presented in the last section.

## 2. Theoretical Background

This section summarizes basic formalisms and concepts for dynamic diagnosis. The notion of time adopted in this paper consists of a discrete sequence of time step, which is derived from the assumption that the system can be viewed as a synchronous transition system [22].

### 2.1. Component and System Model

Supposed that the system to be diagnosed has *n* individual components, the component model can be built as a tuple:

(1) $$G_{i}=(CV,CS,\delta )$$

where:

- CV (component variable) is a set of variables for component *i*. It can be partitioned into mode variables, command variables and attribute variables. Mode variables define the possible behavioral modes for component. Command variables are the external controlled signals. Attribute variables include inputs, outputs and any other variables used to define the behavior of the component.
- CS (component constraint) is a set of formulas constraints, which consists of mode constraints and other constraints. Mode constraints define the physical behavior in certain mode. Other constraints denote the remaining unchanged constraints *i.e*., structure constraints.
- δ (transition relation) is denoted as a tuple $\left(S_{t},S_{t+1},Guard,Prob\right)$ from time *t* to time *t* + 1. $S_{t}$ and $S_{t+1}$ are the mode assignment at time *t* and *t* + 1, respectively. *Guard* is the transition event. Some events are observable (e.g., commands issued from external actuator), while the rest are unobservable (e.g., autonomous or fault events). *Prob* is a transition probability from $S_{t}$ to $S_{t+1}$.

The overall system model *G* is modeled as a composition of synchronous operation on components. Formally, we can give definition of system model as follows:

(2) $$G=(G_{1}\cup G_{2}\cup ...\cup G_{n},SV,SC)$$

where $G_{1},G_{2},...,G_{n}$ denote the *n* component model of the system. SV (system variable) is a set of system I/O variables, which establishes the interconnection with the outside world. SC (system constraint) is a set of constraints that captures the interconnections among components.

### 2.2. Simulation-Based Dynamic Diagnosis

In this subsection, we firstly give the formal definition of static diagnosis, and then extend it into simulation-based dynamic diagnosis. For static systems, the aim of diagnosis problem is to check the joint consistency between observation and a set of constraints including component constraints and system constraints [27]. Since the constraints for each component depend on certain nominal or fault behavior mode, the formal definition of static diagnosis can be given as follow:

(3) y∪stateconstraints(mode)|≠⊥

where *y* is the observation. State constraints stateconstraints(mode) are composed of the component constraints in particular behavior mode mode and system constraints.

If the observations are available not just for one snapshot of system behavior, but for a whole observation period, the diagnosis for static systems will be extended into dynamic systems. The behavior model for dynamic systems not only has the state constraints, but also the relations between states across time:

(4) dynamicmodel(mode)=stateconstraints(mode)∪temporalconstraints(mode)

According to the definition of component model, the relations between states temporalconstraints(mode) correspond to the transition relation δ. Therefore, the dynamic diagnosis must check the individual observation snapshot for consistency with the state constraints, and satisfy the restriction of the pairs of adjacent states, which can be denoted as:

(5) y∪stateconstraints(mode)∪temporalconstraints(mode)|≠⊥

Formally, the definition of simulation-based dynamic system is modeled as follow:

(6) $$SBDS=(G,B_{0},\sigma )$$

where:

- G is an entire system model.
- $B_{0}=(S_{1,0},S_{2,0},...,S_{n,0})$ is the initial belief state, which is constituted by the mode for each component at time step 0.
- σ is a observation sequence $\left(y_{0},y_{1},...,y_{t},...y_{l}\right)$, where $y_{t}$ are the observation variables or command variables at time step t.

On the basis of Equations (5) and (6), the aim of simulation-based dynamic diagnosis is to find the system state $B_{t}$ at each time step *t*, which is consistent with observation $y_{t}$, and is transitioned from $B_{t-1}$.

### 2.3. Classical Belief State Update

A well known problem of model-based diagnosis is that the number of possible trajectories will become so large to be unmanageable as the system evolves. Typically, the number of trajectory hypotheses is exponential in the number of discrete modes and time steps considered. A general solution is the introduction of a preference criterion among belief states.

Most of the preference criteria are based on quantitative probability. A belief state is a probability distribution over the states of a system, which represents the likelihood of the system in any single state given observation and command sequence. Assuming that the system is Markovian, the belief state is then computed using standard Hidden Markov Model (HMM) belief state update equations:

(7) $$\begin{array}{ll} P(B_{t+1}^{j}|y_{1:t+1}) & =P(B_{t+1}^{j}|y_{1:t},y_{t+1})\;=\alpha P(y_{t+1}|B_{t+1}^{j})P(B_{t+1}^{j}|y_{1:t})\\ \; & =\alpha P(y_{t+1}|B_{t+1}^{j})\sum_{B_{t}^{i}}P(B_{t+1}^{j}|B_{t}^{i})P(B_{t}^{i}|y_{1:t}) \end{array}$$

where α denotes the normalization term. This process is known as recursive estimation [28]. It includes two steps: *Prediction*, in which given a current belief state prior distribution $P(B_{t}^{i}|y_{1:t})$, belief state estimator selects a possible transition model $P_{T}\text{=}P(B_{t+1}^{j}|B_{t}^{i})$ to predict a new state; *Update*, in which the predicted state is compared with an observation model $P_{O}=P(y_{t+1}|B_{t+1}^{j})$ to adjust the probability.

For simulation-based dynamic diagnosis, the Prediction step uses transition relation temporalconstraints(mode) to propagate the system dynamics into the future by only considering the current belief state and commands, while the Update step evaluates the estimates by checking the consistency between observation y and state constraints stateconstraints(mode). In this paper, the observation probability distribution $P_{O}=P(y_{t+1}|B_{t+1}^{j})$ is defined using a consistency approach similar to the Livingstone [21,22], and can be calculated as follow:

(8) $$P_{O}=P(y_{t+1}|B_{t+1}^{j})=\{\begin{array}{c} 1\;if\;stateconstraints(B_{t+1}^{j})|=y_{t+1}\\ 0\;if\;stateconstraints(B_{t+1}^{j})|\neq y_{t+1} \end{array}$$

## 3. Exploitation of LUG for State Tracking and Fault Diagnosis

A planning graph represents a relaxed look-ahead of the belief state space that identifies propositions reachable at different depths. It can be typically described as layered graphs of vertices $\left(P_{0},A_{0},..,A_{t-1},P_{t},A_{t},...\right)$, where each level *t* consists of a proposition layer $P_{t}$ and an action layer $A_{t}$. More specifically, proposition layer $P_{t}$ denotes the set of propositions at level *t*, while action layer $A_{t}$ includes all actions that have all of their precondition propositions in $P_{t}$. Edges between the layers describe the propositions in action preconditions (from $P_{t-1}$ to $A_{t-1}$) and effects (from $A_{t-1}$ to $P_{t}$) [29].

LUG, proposed by Bryce [25,29,30], extends traditional planning graph in the following two ways. Firstly, since uncertainty is considered in Bryce’s approach, an extra effect layer $\varepsilon_{t+\Delta }$ is introduced into each level *t*, where Δ→0. An effect is in the effect layer $\varepsilon_{t+\Delta }$ if its associated action is in the action layer $A_{t}$ and every one of its antecedent propositions is in $P_{t}$. As a result, the uncertainty planning graph can be denoted as a sequence of layers $\left(P_{0},\varepsilon_{0+\Delta },A_{0},..,A_{t-1},\varepsilon_{(t-1)+\Delta },P_{t},A_{t},...\right)$. Secondly, LUG implicitly represents multiple uncertainty planning graphs by collapsing the graph connectivity into one uncertainty planning graph and uses annotations, called labels, to retain information about multiple worlds. Bryce has successfully applied this data structure to solve the probabilistic conformant planning problem with actions whose effects are uncertain.

The probabilistic conformant planning problem is closely related to state tracking and fault diagnosis of discrete systems [31], because the task of generating most likely belief states to match given observations can be viewed as a probabilistic plan generation problem. In particular, both two problems exhibit many similar features, such as finite state space, the uncertainty of the initial state and action effects, reachable goals and so on. Therefore, LUG is introduced to represent dynamic diagnosis process of discrete systems. Proposition layer, action layer and effect layer in LUG correspond to the possible belief states, transition events and the possible results of transition in simulation-based dynamic diagnosis.

In order to address the exponential increasing problem of possible belief states, the Monte Carlo (MC) technique [32] is employed to sample belief states in our method. First, unlike the traditional method using exact quantitative probability, we turn to approximation of probability by means of particles. The number of particles in a particular belief state represents the likelihood of this belief state. This approximate strategy allows our approach to focus on the highly probable belief states, without checking a prohibitively large number of unlikely belief states. Second, the MC technique in this paper is only used for sampling, and particles are assumed to be unit weight throughout simulation. Unlike generic particle filter, our work does not use observations to weight particles for re-sampling. For instance, assuming that belief state $B_{1}$ has 100 particles and a transition occurs from belief state $B_{1}$ to belief state $B_{2}$ with probability 0.95, there will be 95 particles in belief state $B_{2}$ after performing this transition. Third, every particle is tagged with unique symbol, which can be used to analyze the system possible evolution trajectories.

**Figure 1:**
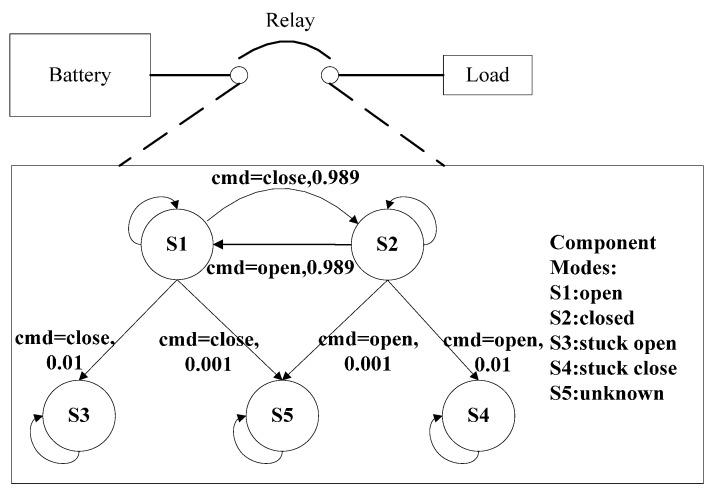
A simple circuit consisting of a battery, relay and a load.

A simple circuit shown in Figure 1 is used as an illustrative example. The relay in this circuit is modeled as an automaton with five discrete modes: S1: open, S2: closed, S3: stuck_open, S4: stuck_closed and S5: unknown. The mode transition is probabilistic. When the initial mode is S1: open with the command close, the possible successor modes include S2: closed, S3: stuck open and S5: unknown with the transition probability 0.989, 0.01 and 0.001, respectively.

**Figure 2:**
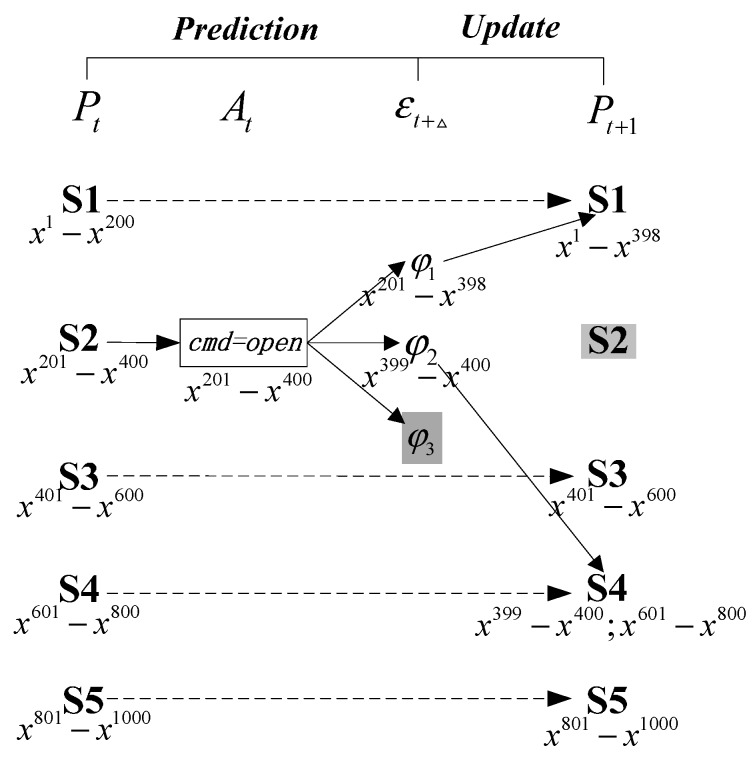
Relay depicted by LUG.

Figure 2 reports the LUG for this relay. The number of particles is set to 1000. Proposition layer $P_{t}$ and $P_{t+1}$ represent the possible belief states at time *t* and *t* + 1. Action layer $A_{t}$ contains the transition events at time *t*. In this layer, controlled events and autonomous events are denoted by propositional logic formula and function, while idle events are drawn as dashed line. The effect layer $\varepsilon_{t+\Delta }$ describes the possible transition effects after an infinitesimally time Δ and depends on the proposition layer $P_{t}$ and action layer $A_{t}$. According to the formal definition of classical belief state update in Section 2.3, the Prediction step in LUG is from proposition layer $P_{t}$ to effect layer $\varepsilon_{t+\Delta }$, and the Update step is between effect layer $\varepsilon_{t+\Delta }$ and proposition layer $P_{t+1}$. The label below each belief state contains the tagged particle $x^{i}$. At time t, 1000 particles are initialized using prior distribution of current belief state. If current mode of relay is unknown, the uniform distribution will be adopted. First, the Prediction step is executed. Take a possible transition relation (S2:closed,S1:open,cmd=open,0.989) for example, proposition logic (S2:closed)∩(cmd=open)⇒(S1:open) is performed, and 200×0.989≈198 particles are transitioned from S2:closed to effect $\varphi_{1}$. After that, the observation at time step *t* + 1 is taken into account. In case that the estimated belief state in effect $\varphi_{1}$ is consistent with observation ($stateconstraints(\varphi_{1})|=y_{t+1}$), all the particles in effect $\varphi_{1}$ are moved further into belief state S1:open. As can be seen from Figure 2, four possible belief states: S1, S3, S4 and S5 with 398, 200, 202 and 200 particles are captured at time-step *t* + 1, and possible system evolution trajectories also can be obtained by the tagged particles.

## 4. Proposed State Tracking and Fault Diagnosis Algorithm

This section presents the dynamic diagnosis process of discrete systems based on LUG in greater detail. First of all, a novel one step look-ahead technique is introduced to capture the fault mode with low likelihood. Moreover, the overall algorithm is described. Finally, the correctness, incompleteness and computational complexity are analyzed from a theoretical view point.

### 4.1. One Step Look-Ahead

**Figure 3:**
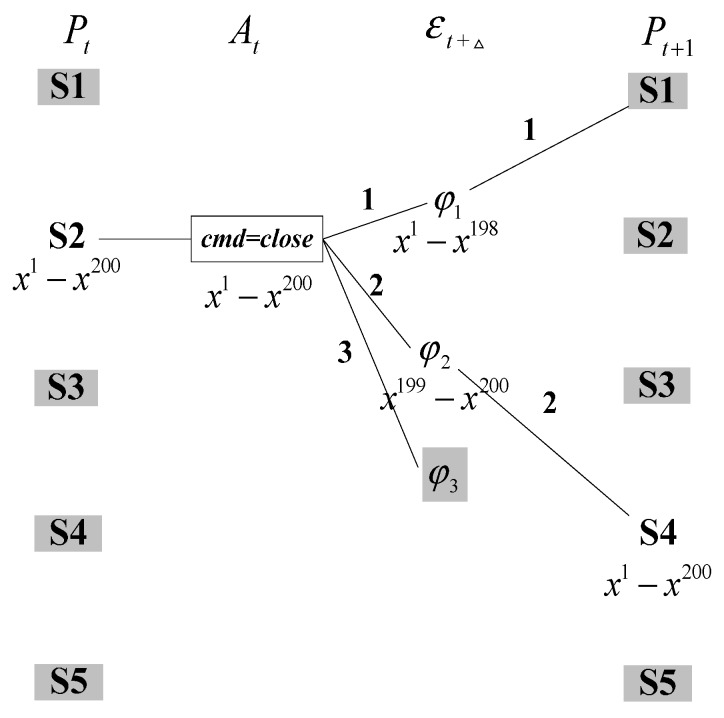
Simple one-step belief state estimation.

As mentioned earlier, this paper introduces unit weight particles to evaluate the likelihood of belief state and filter out a prohibitively large amount of unlikely belief states. However, unit weight particles will bring a serious problem called sample impoverishment. Now the relay example is also used to describe this situation. To simplify the problem, belief state estimation in a single time-step is illustrated by using one initial belief state S2 with 200 particles. As can be seen from Figure 3, Prediction step is firstly executed to generate two possible transition results: $\varphi_{1}$ with 198 particles and $\varphi_{2}$ with two particles. Effect $\varphi_{3}$ is discarded, because it cannot be assigned a particle with the low transition probability 0.001. Then, *Update* step uses current observation to update belief state distribution. If successor belief state S1 at time *t* + 1 is in conflict with observation, only S4 will occupy all the 200 particles after normalization. In this case, classical method leads to losing the possible solution S5 resulted from effect $\varphi_{3}$. The reason is that fault events usually have a very low prior probability. When a system is in its normal condition, the high probability transition results are consistent with observations, so only the solutions with low likelihood are removed. However, once a fault occurs, maybe no particles can transition into fault state. Therefore, the real fault cannot be reliably detected.

To tackle sample impoverishment problem, our proposed algorithm adopts a novel strategy that combines prior transition probability and observation information to choose the most likely successor belief states. In particular, best-first A* search [33] is employed to recursively calculate the *a posteriori* transition probability:

(9) $$P_{R}^{i}=\alpha \times P_{O}^{i}\times P_{T}^{i}$$

where $P_{O}^{i}$ and $P_{T}^{i}$ denote the observation probability and the prior probability for transition *i*, respectively. Once all the particles can be assigned according to a set of generated *a posteriori* transition probability $\left\{P_{R}^{1},P_{R}^{2},...,P_{R}^{i}\right\}$, the search process will be terminated.

Unfortunately, computing the normalization term $\alpha =1/\left(\sum_{j=1}^{n}P_{O}^{j}\times P_{T}^{j}\right)$ exactly is intractable. Therefore, we transform this equation into another form, and then employ an approximate strategy to converge the real value:

(10) $$\begin{array}{ll} \alpha & =1/\left(\sum_{j=1}^{n}P_{O}^{j}\times P_{T}^{j}\right)=1/\left(\sum_{j=1}^{m}P_{O}^{j}\times P_{T}^{j}+\sum_{j=m+1}^{n}P_{O}^{j}\times P_{T}^{j}\right)\\ \; & =1/\left(\sum_{j=1}^{m}P_{O}^{j}\times P_{T}^{j}\right)=1/\sum_{j=1}^{m}P_{T}^{j}=1/\left(1-\sum_{j=m+1}^{n}P_{T}^{j}\right) \end{array}$$

In Equation (10), all the possible transitions j=[1,n] are broken up into two parts: j=[1,m]∪[m+1,n], where [1,m] denotes the consistent transitions whose successor belief states are entailed with observation ($P_{o}=1$), and [m+1,n] describes the inconsistent transitions whose successor belief states are refuted by the observation ($P_{o}=0$). Since it is hard to obtain all the inconsistent transitions [m+1,n] in each time-step, the normalization term can be approximated as follows:

(11) $$\alpha :=1/\left(1-\sum_{j=1}^{l}P_{T}^{j}\right)$$

where l denotes a set of inconsistent transitions that have been generated during the enumeration process. Note that the normalization term needs to be recalculated, once a new inconsistent transition is determined.

**Table 1:** Estimation process using look-ahead technique.

Loop	Path	$P_{T}$	$P_{O}$	$P_{R}$	Number of Particles
1	S2→S1	0.989	0	0	0
2	S2→S4	0.01	1	0;0.91	182
3	S2→S5	0.001	1	0;0.91;0.09	18

Table 1 shows the estimation process of the relay example using look-ahead technique, where $P_{R}$ is a list to preserve all the obtained *a posteriori* transition probability. In first loop, path S2→S1 is generated. Since it is an inconsistent path, the *a posteriori* transition probability $P_{R}^{1}$ is set to 0. Second loop analyzes path S2→S4, which is consistent with the observation. The *a posteriori* transition probability for this path is calculated as $P_{R}^{2}=0.01/(1-0.989)\approx 0.91$. After that, we continue to generate the third path S2→S5, because these two expanded paths cannot occupy all the 200 particles. Although the prior transition probability for this path is only 0.001, the *a posteriori* transition probability increases to $P_{R}^{3}=0.001/(1-0.989)\approx 0.09$. Since 200 particles can be assigned using generated *a posteriori* transition probability list (0; 0.91; 0.09), the search is terminated. Correspondingly, belief state S4 and S5 own 182 and 18 particles. We see that our proposed algorithm can capture an additional belief state S5, when compared to the traditional method.

### 4.2. Description of the Approach

Overall, our state tracking and fault diagnosis approach for dynamic systems are composed of two main steps:

- A fast roll forward process that uses the forward propagation to extract the likely belief states at each time-step.
- A quick roll back process using tagged particles to generate the possible trajectories.

The roll forward process is shown in Algorithm 1. Since the procedures have been described in Section 4.1, we will not provide more explanations.

Algorithm 2 describes the roll back process to generate the possible trajectories $Trajectory=\{T_{1},T_{2},...,T_{n}\}$, where trajectory $T_{n}=(Traj_{n},w_{n})$ can be defined as $Traj_{n}=\{B_{0},A_{0},B_{1},A_{1},B_{2},...\}$ with corresponding weight $w_{n}$. The key idea is to back-propagate using the serial number of each particle. In particular, if belief state $B_{t-1}$ and belief state $B_{t}$ at adjacent time-steps capture the particles with the same serial number, a trajectory can be constructed between belief state $B_{t-1}$ and belief state $B_{t}$, and the weight w for this trajectory is the number of particles shared by both belief state $B_{t-1}$ and belief state $B_{t}$. This algorithm is usually executed to analyze and evaluate the evolution history of the system, once the faults are detected.

**Algorithm 1**: Roll forward process

1: **Input**: Initial belief state $B_{0}^{1},...,B_{0}^{n}$; Number of the particles N
2: **Output**: LUG with the most likely belief state $B^{0},...B^{i}$ at each time step
3: Sample N particles using the prior probability distribution $P(B_{0})$
4: Add the initial belief state $B_{0}^{1},...,B_{0}^{n}$ to proposition layer $P_{0}$
5: **For** each time-step *t* >0 **do**
6:    **For** each belief state $B_{t}^{i}$ in $P_{t}$ **do**
7:      **If** all the particles can be assigned according to a set of obtained *a posteriori* transitions
             probability $\left\{P_{R}^{1},P_{R}^{2},...\right\}$ **Then** break
8:      Execute possible transitions $k:=B_{t}^{i}\to B_{t+1}^{j}$ and store the corresponding effect $\varphi_{k}$ into $\varepsilon_{t+\Delta }$
9:      **If** the successor belief state $B_{t+1}^{j}$ is consistent with observation $y_{t}$
10:          Save the belief state $B_{t+1}^{j}$ into proposition layer $P_{t+1}$
11:          Calculate the *a posteriori* transitions probability $P_{R}^{k}$
12:          Insert $P_{R}^{k}$ into a set of obtained *a posteriori* transitions probability $\left\{P_{R}^{1},P_{R}^{2},...\right\}$
13:      **Else**
14:          Recalculate the normalization term α
15:          Update the set of obtained *a posteriori* transitions probability $\left\{P_{R}^{1},P_{R}^{2},...\right\}$
16:      **End If**
17:    **End For**
18:  Assign the particles for the belief state $B_{t+1}$ in $P_{t+1}$ according to a set of obtained *a posteriori* transitions probability $\left\{P_{R}^{1},P_{R}^{2},...\right\}$
19: **End For**



**Algorithm 2**: Roll back process

1:  **Input**:    Label uncertainty graph LUG
2:  **Output**: A set of possible trajectories $Trajectory=\{T_{1},T_{2},...,T_{n}\}$
3:  **For** each time-step *t*>0 **do**
4:         **For** each belief state $B_{t}$ in proposition layer $P_{t}$ **do**
5:               **For** each particle $p_{j},j=1,...,N$ in belief state $B_{t}$ **do**
6:                     Extract the belief state $B_{t-1}$ in $P_{t-1}$ which also contains the same particle $p_{j}$
7:                     Roll back to generate the trajectory $Traj_{j}=\{B_{t-1},A_{t-1},B_{t}\}$ from $B_{t}$ to $B_{t-1}$
8:                     Construct a new trajectory tuple $T_{j}=(Traj_{j},w_{j}=1)$
9:                     Add $T_{j}$ into obtained most likely trajectories Trajectory
10:                   Merge the same trajectory and update the weight 
11:               **End For**
12:      **End For**
13: **End For**



**Figure 4:**
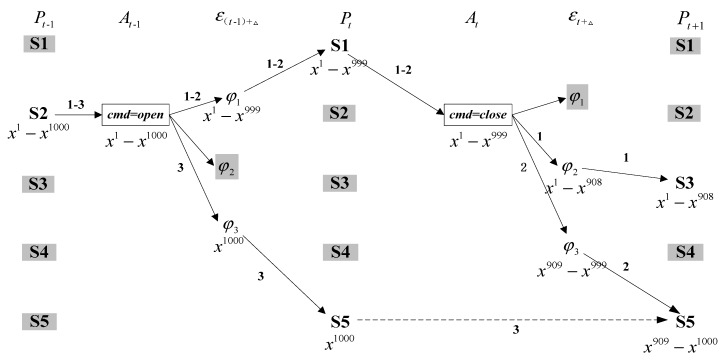
Two time-step state estimation using LUG for relay.

The simple relay model is again considered as an example to further describe the combination of roll forward and roll back process. Assumed that only S2 with 1000 particles is available at time *t* − 1, Command open is issued at time *t* − 1. At time t, S1 and S5 are consistent with observation. After executing command close at next time step, S3 and S5 match with measurement. Figure 4 shows the LUG structure for relay at these two successive time steps. It is easy to see that the probability of belief state S3 and S5 at time *t* + 1 are 90.8% and 9.2%. Three different evolution trajectories can be rolled back to obtain as Trajectory1 = {S2, open, S1, close, S3}, Trajectory2 = {S2, open, S1, close, S5} and Trajectory3 = {S2, open, S5, close, S5} with the probability 90.8%, 9.1% and 0.1%, respectively.

### 4.3. Analysis of the Approach

#### 4.3.1. Correctness and Incompleteness

Since the proposed approach runs for a whole observation period to track and diagnose the system, we should prove the correctness of the *Prediction* step and *Update* step at each single time-step. As mentioned earlier, the aim of the *Prediction* step is to estimate the system state at the next time-step based on the current belief state, commands and temporal constraints, while the *Update* step checks the consistency between observations and state constraints in the estimated system state. Struss and Dressler [34] derived a correctness result for the consistency test in a static system. This result is relevant to the present approach, because it guarantees the correctness of the *Update* step. In this subsection, we extend this analysis to our approximate simulation-based dynamic diagnosis.

For the *Prediction* step, the proposition logics corresponding to the transition relation in each component model are performed to reason the possible successor modes. Without loss of generality, all the possible transition results in our proposed approach can be divided into three disjoint classes:

- Case 1: the effect $\varphi_{i}$ can be assigned more than one particle according to the prior transition probability $P_{T}$, and the observation is consistent with successor belief state (See path 2 in Figure 3).
- Case 2: the effect $\varphi_{i}$ can also be distributed more than one particle, but the observation refutes the successor belief state (See path 1 in Figure 3).
- Case 3: the effect $\varphi_{i}$ cannot be assigned one particle using the prior transition probability $P_{T}$ (See path 3 in Figure 3).

For Case 1 and Case 2, these possible successor modes can be assigned more than one particle, and may be kept or dropped after the consistency test. In terms of Case 3, since these successor modes usually have low prior transition probability, the number of the remaining particles determines whether the proposed approach needs to check the consistency for these modes. Actually most of the probability space can be covered by just a few modes in the state estimation of discrete systems. If all the obtained belief distribution can reach the estimation accuracy determined by a predefined number of particles, all the remaining low likelihood modes can be discarded.

On the basis of the above analysis, the present approach is more efficient than using a consistency test for every mode, and achieves a close enough approximation. Moreover, it can be implemented as an anytime algorithm, and the trade-off between accuracy and time efficiency can be achieved by varying the number of particles. Finally, this analysis process also reveals the correctness and incompleteness of our approach.

#### 4.3.2. Complexity

In this subsection, the complexity in a single time-step will be analyzed. Previously, we assumed that the system to be diagnosed is modeled as *n* concurrent individual components with *b* possible successor modes, and the number of particles is *p_num*. In addition, the computational complexity for a single consistency test is evaluated as a constant *C* to simplify the problem.

Martin *et al*. [23] analyzed the complexity of best-first A* search for a single initial state, and concluded that the best case complexity is roughly n×b and the worst case complexity is $b^{n}$. In terms of the roll forward process, since the number of all the possible successor modes is $b^{n}$ in a single time-step, all the particles transition into the successor mode with the lowest prior probability in the worst case, and the complexity is roughly $O(b^{n}+b^{n}\times C)$. In the best case, all the particles transition into the first possible successor mode, so the complexity is O(n×b+C). For the roll back process, the worst case has two different conditions: (1) all the particles exist in different modes ($p\_num<b^{n}$); or (2) each possible successor mode captures more than one particle ($p\_num>b^{n}$). Considering these two cases together, the complexity of this transversal process is $O(\min(p\_num,b^{n}))$. On the other hand, the best case complexity is also O(1) when all the particles are in a single mode. As a summary, Table 2 shows the complexity for our proposed algorithm.

**Table 2:** The complexity for our proposed algorithm.

	Best Case	Worst Case
Roll forward process	O(n×b+C)	$O(b^{n}+b^{n}\times C)$
Roll back process	O(1)	$O(\min(p\_num,b^{n}))$

## 5. Experimental Results

**Figure 5:**
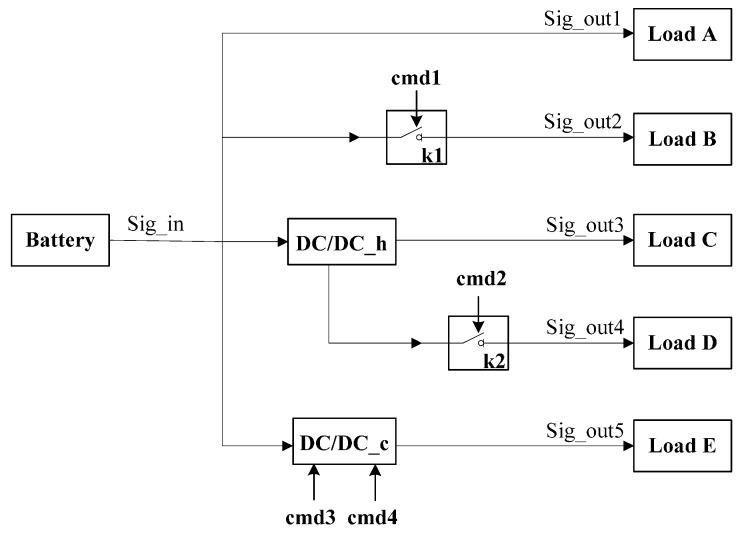
Selected subset of the power supply control unit.

We apply our state tracking and fault diagnosis approach on a simulation model of a real-world system—a selected subset of the power supply control unit of a spacecraft. This subsystem, shown in Figure 5, consists of an input Sig_in from a battery and five outputs: (1) output Sig_out1 directly connected to Load A; (2) output Sig_out2 connected to Load B that is controlled by relay K1; (3) output Sig_out3 connected to Load C that is controlled by hot backup DC/DC module (DC/DC_h); (4) output Sig_out4 connected to Load D that is controlled by both hot backup DC/DC module and relay K2; and (5) output Sig_out5 connected to Load E that is controlled by cool backup DC/DC module (DC/DC_c). An external actuator issues commands cmd1, cmd2, cmd3 and cmd4 to control the relay K1, K2 and cool backup module. In our experiment, six sensors are used to collect observations: system input: Sig_in and system outputs: Sig_out1, Sig_out2, Sig_out3, Sig_out4 and *Sig_out5*.

**Figure 6:**
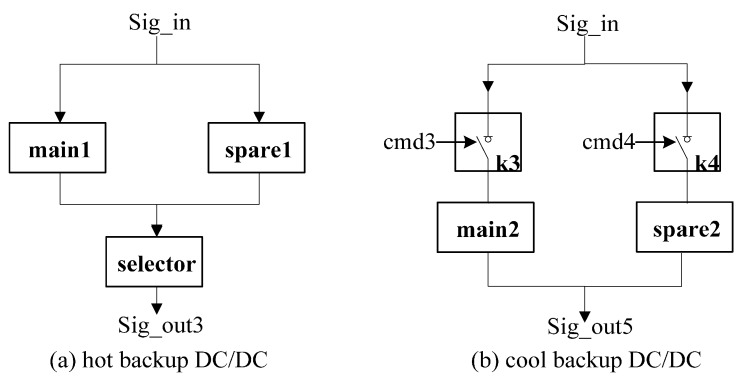
DC/DC module.

The schematics of the hot backup DC/DC module and cool backup DC/DC module are presented in Figure 6. Four components main1, main2, spare1 and spare2 are voltage converting units. Figure 6a shows the hot backup DC/DC module. The function is that component selector selects the voltage with higher value from main1 and spare1 to output. In the cool backup DC/DC module (see Figure 6b), the external commands cmd3 or cmd4 switch relays K3 and K4 and determine the output voltage.

This selected subset of the power supply control unit involves nine components: four voltage converting units, four relays and one selector. More specifically, the voltage converting unit has five different discrete modes: nominal (M1), overvoltage protection (M2), overvoltage protection failure (M3), voltage conversion failure (M4) and unknown mode (M5). Table 3 gives the mode transition matrix for this component. In addition, the relays and selector also contain five discrete modes. For lack of space, the transition matrixes for these components are not shown in this paper. Therefore, we can calculate that the system can potentially operate in roughly $5^{9}=1953125$ distinct modes at each time-step, and the full system trajectories will even reach up to $1953125^{t}$ as the system evolves.

**Table 3:** The transition matrix for voltage converting unit.

Source Mode	Transition Constraint	Possible Successor Modes				
		M1	M2	M3	M4	M5
M1	sig_in < 97	0.989	0	0	0.01	0.001
M1	sig_in >= 97 sig_in <= 103	0.979	0	0	0.02	0.001
M1	sig_in > 103	0	0.959	0.02	0.02	0.001
M2	sig_in < 97	0.989	0	0	0.01	0.001
M2	sig_in >= 97 sig_in <= 103	0.979	0	0	0.02	0.001
M2	sig_in > 103	0	0.959	0.02	0.02	0.001
M3	-	0	0	1	0	0
M4	-	0	0	0	1	0
M5	-	0	0	0	0	1

Several groups of simulations were conducted on a test set, which includes the nominal scenario and the occurrence of a fault in one, two components and three components at the same time. The experimental results refer to a C++ implementation of the diagnostic algorithm using a personal computer featuring an Intel (R) Core (TM) i3 CPU with 2.27 GHz, 4GB RAM (Lenovo, Kunshan, China), and are presented in the following subsections.

### 5.1. Basic Results

The aim of these simulations is to evaluate the space and time performance results of our state tracking and fault diagnosis method. For these simulations, nominal, single fault, two faults and three faults are considered, and the number of particles is set to 500.

The good experimental time complexity results are confirmed by looking at the computational cost in terms of CPU time. Table 4 reports the average and the maximum CPU time for single-step mode estimation. The average time increases when more faults are injected. However, the CPU time is very low with three faults so that we claim that the algorithm can perform on-line.

For the belief state search problem, the number of expanded nodes is used to measure the space performance of algorithms. Moreover, since the consistency function usually consumes plenty of computing resources, the so-called times of consistency function are also employed to qualitatively evaluate the time performance. On the basis of the above consideration, the average and maximum number for these two values are also evaluated in Table 5. As expected, these two values will increase slowly as more faults are considered, and generally reach a maximum value at the fault detection time, because a large amount of nodes are expanded to check the consistency with observation at that time.

**Table 4:** Time statistics with single-step mode estimation (confidence 95%).

Scenario	Average Time (ms)	Max Time (ms)
Nominal	29.725 ± 0.634	85.46
Single Fault	67.873 ± 1.770	143.68
Double Faults	93.661 ± 5.198	328.65
Three Faults	103.759 ± 6.866	423.57

**Table 5:** The sizes of expanded nodes and the called times of consistency function per time step (confidence 95%).

Scenario	Expanded Nodes		Called Times of Consistency Function	
	Average Number	Max Number	Average Number	Max Number
Nominal	96.538 ± 1.6221	116	8.2000 ± 0.1384	18
Single Fault	103.455 ± 2.8798	151	14.4000 ± 0.6728	46
Double Faults	108.727 ± 3.0792	202	22.7000 ± 1.8675	110
Three Faults	115.545 ± 5.3045	273	24.5000 ± 2.1935	128

### 5.2. Number of Particles

**Figure 7:**
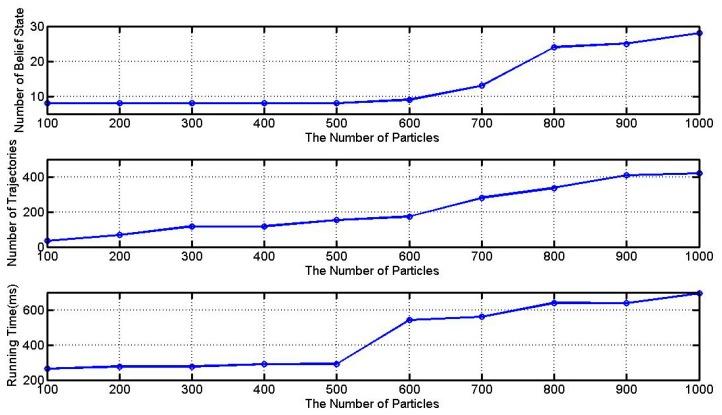
Effect of the number of particles.

In this subsection, we conduct a set of simulations in the nominal scenario with 10 time-steps to test the sensitivity of the number of particles to the performance of our approach. The number of particles varies from 100 to 1000 and typical experimental results are shown in Figure 7. As can be seen from this figure, the performance of our method is relevant to the number of particles. As the number of particles increases, more belief states and trajectories are obtained, and the time consumption also goes up.

### 5.3. Comparison with Other Algorithms

We now compare the performance of our approach with two *k* best methods: (1) *k* best BFTE algorithm and (2) *k* best CDA* algorithm with respect to the following aspects: (1) estimation accuracy; (2) the consumed time as the number of obtained belief states increases; and (3) the sensitivity of different approaches’ performance to estimation time steps.

**Figure 8:**
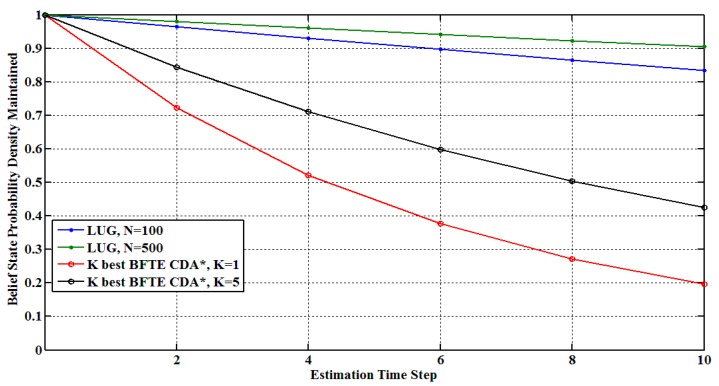
Probability density maintained over time.

As discussed earlier, *k* best methods choose *k* best trajectories or belief states to track system dynamics, and the value of *k* determines their estimation accuracy and performance. Blackmore *et al*. [35] pointed out that their estimation accuracy depends on whether or not *k* is large enough for real belief state distribution. In other words, when the distribution over belief state is relatively flat, *k* best methods maybe lead to losing the solution. Compared to these methods, our approach is robust for this situation. Generally speaking, the number of particles directly determines the estimation accuracy in our approach. Assumed that 100 particles are used, the loss of belief state probability density is less than 1% at each time step. If the particles increase to 500 or 1000, the loss will reduce to less than 0.2% or even 0.1%. Therefore, the number of obtained belief states at each time-step is dynamic adaptive and critically dependent on current belief state distribution. For a relatively concentrated distribution, our algorithm just needs to calculate a smaller number of belief states. On the other hand, more belief states will be obtained, when the desired distribution is relatively flat. Figure 8 shows the maintained belief state probability density over many cycles. Since the *k* best CDA* algorithm only improves computational performance but not estimation accuracy when compared to *k* best BFTE algorithm, only *k* best CDA* algorithm is shown in this figure. It is easy to find that the reduction in probability density is exponential in the number of time steps for both LUG and *k* best algorithm, but the rate of decay is clearly slow for our proposed method.

In second experiment, we investigate a set of simulations with 10 time-steps to show the time consumption of different algorithms varying predefined parameter. In Table 6, $N_{P}$, $N_{B}$ and $N_{T}$ denote the number of particles, belief states and trajectories, respectively. It is easy to see that *k* best CDA* algorithm has a better time performance than the *k* best BFTE algorithm. Moreover, the difference between the proposed approach and *k* best CDA* algorithm can be analyzed in case that the same number of belief states $N_{B}$ or trajectories $N_{T}$ are obtained. When the *k* value is smaller than 3, the time performance of *k* best CDA* algorithm is good enough. However, when the *k* value is set to 10 ($N_{T}=10$), *k* best CDA* algorithm captures four belief states, but the time result is 4185.69 ms. On the other hand, the proposed method ($N_{p}=100$) can captures eight belief states, and only consumes 263.87 ms. Therefore, the proposed method achieves more estimation accuracy and consumes less time, and this advantage becomes significantly apparent as the number of obtained belief states or trajectories increases.

**Table 6:** The time consumption of different algorithms (confidence 95%).

LUG				BFTE			CDA*		
$N_{P}$	$N_{B}$	$N_{T}$	Time (ms)	$N_{T}$	$N_{B}$	Time (ms)	$N_{T}$	$N_{B}$	Time (ms)
100	8	35	263.87 ± 0.21	1	1	51.97 ± 0.08	1	1	27.38 ± 0.03
200	8	67	276.70 ± 0.25	2	2	156.89 ± 0.12	2	2	82.15 ± 0.05
300	8	117	277.38 ± 0.32	3	3	489.86 ± 0.43	3	3	194.76 ± 0.45
400	8	117	289.23 ± 0.47	4	3	809.56 ± 0.54	4	3	375.23 ± 0.49
500	8	152	292.08 ± 0.63	5	3	1352.88 ± 0.61	5	3	587.18 ± 0.58
600	9	174	541.17 ± 0.67	6	3	2307.51 ± 0.65	6	3	961.42 ± 0.69
700	13	280	559.83 ± 0.71	7	3	3573.87 ± 0.73	7	3	1276.36 ± 0.76
800	24	337	640.24 ± 0.77	8	3	4922.32 ± 0.82	8	3	2058.53 ± 0.71
900	25	408	638.71 ± 0.81	9	3	6214.18 ± 1.03	9	3	3468.74 ± 0.92
1000	28	419	692.72 ± 0.85	10	4	8446.02 ± 1.15	10	4	4185.69 ± 0.97

**Figure 9:**
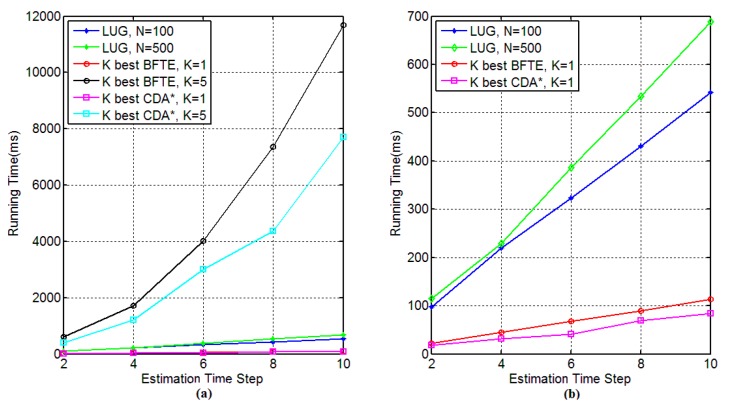
The performance results for different time step.

Figure 9 shows the performance results as the estimated time-step increases for the third experiment. The number of particles in our approach is set to 100 and 500, while both BFTE and CDA* consider the value of *k* as 1 and 5 together. As can be seen from Figure 9a, the time consumption of BFTE and CDA* with *k* = 5 increase sharply, and the other curves go up smoothly. Since Figure 9a cannot clearly show the differences among our approach, BFTE and CDA* with single-estimation, Figure 9b zooms in these curves. This figure shows that our approach with 500 particles has more time consumption than 100 particles. It is in line with our previous analysis in Section 5.2. Similarly, we can also see that single-estimate results for BFTE and CDA* outperform our approach.

As a summary, *k* best BFTE and CDA* algorithm are well suited for a system with a relatively concentrated belief state distribution, while our approach can be applied for the systems with either concentrated or flat distributions. Moreover, our approach has better estimation accuracy and outperforms the *k* best BFTE and CDA* algorithms for sufficiently sized belief states.

## 6. Conclusions

In this paper, we propose a novel simulation-based fault diagnosis approach, which models the systems as concurrent probabilistic automata and applies LUG to state tracking and fault diagnosis of these systems. Moreover, the MC technique is introduced into this scheme, so our algorithm is anytime, and can balance between accuracy and time efficiency by varying the number of particles. On the one hand, the particles control the breadth of best-first A* search and maintain most likely belief states; on the other hand, the tagged particles can be used to generate system evolution trajectories. Finally, this paper analyzes the sample impoverishment problem resulted from the MC technique, and employs a novel recursively one step look-ahead strategy to mitigate this situation and improve the estimation accuracy.

The method has been successfully applied to a non-trivial real-world example: a power supply control unit of a spacecraft. The experimental results show its satisfactory performance including estimation accuracy, time and space complexity. It is also possible to diagnose the system without making any simplifying assumption such as single fault. In future work, we will introduce some variance into our predefined probability transition matrix, because the fixed transition probability in our experiment is relatively simple. Moreover, distributed diagnosis techniques can efficiently decrease the computational complexity for large-scale complex systems, so this is another research direction for the future.
